# Interplay of socioeconomic status, cognition, and school performance in the ABCD sample

**DOI:** 10.1038/s41539-024-00233-x

**Published:** 2024-03-11

**Authors:** Lara Langensee, Theodor Rumetshofer, Johan Mårtensson

**Affiliations:** Department of Clinical Sciences Lund, Division of Logopedics, Phoniatrics and Audiology, Lund University, Scania, Sweden

**Keywords:** Education, Human behaviour

## Abstract

Coming from a disadvantaged background can have negative impact on an individual’s educational trajectory. Some people however seem unaffected and cope well with the demands and challenges posed by school education, despite growing up in adverse conditions, a phenomenon termed *academic resilience*. While it is uncertain which underlying factors make some people more likely to circumvent unfavorable odds than others, both socioeconomic status (SES) and cognitive ability have robustly been linked to school performance. The objective of the present work is to investigate if individual cognitive abilities and SES interact in their effect on grades. For this purpose, we analyzed SES, cognitive, and school performance data from 5001 participants from the *Adolescent Brain Cognitive Development* (ABCD) Study. Ordinal logistic regression models suggest similar patterns of associations between three SES measures (parental education, income-to-needs ratio, and neighborhood deprivation) and grades at two timepoints, with no evidence for interaction effects between SES and time. Parental education and income-to-needs ratio were associated with grades at both timepoints, irrespective of whether cognitive abilities were modeled or not. Neighborhood deprivation, in contrast, was only a statistically significant predictor of reported grades when cognitive abilities were not factored in. Cognitive abilities interacted with parental education level, meaning that they could be a safeguard against effects of SES on school performance.

## Introduction

We may all be born with a blank slate, but that does not imply that we are all born equal. Numerous studies, across different age ranges and cultures, have found links between a person’s socioeconomic background and how well they do in school, indicating that an individual’s educational success is at least in parts determined by where they come from refs. ^1–3^. This effect that has been demonstrated as early as when children only just start attending school^4^, though there is also evidence that this pattern may not be present in developing countries^5^. School performance is not the only facet of development that has been associated with socioeconomic variables. For example, low socioeconomic status (SES) during childhood and adolescence has also been linked with an increased risk for various mental health and behavioral issues^6–11^ while higher SES has been found to relate to higher quality of life and a healthier lifestyle^12,13^. A large population-based study in the UK concluded that associations between a family’s socioeconomic status and their children’s performance in primary school have existed for nearly a century and that the strength of these relationships has remained steady throughout that time^14^, in line with a recent meta-analysis that corroborated the same trend when reviewing over 300 empirical studies from over 100 countries^15^. In contrast, in China the strength of association between SES and academic achievement was found to have gradually decreased during the last couple of decades^16^. An earlier meta-analytic study that reviewed data from more than 100,000 individuals found medium to strong correlations between different indices of students’ SES and their academic achievement^3^.

The influence of additional relevant variables has been studied to create a more nuanced understanding of the relationship between SES and educational attainment. A major influencing factor which has previously been linked to both socioeconomic status and academic achievement are cognitive abilities, the development and refinement of which is an important developmental milestone during childhood. Cognitive functioning refers to mentally storing, retrieving, and processing information and includes a range of skills, for example, abstract reasoning, memory, executive function, attention, language, and processing speed^17–19^. Many of these have been explored with respect to their potential impact on the relationship between SES and attainment in school. For instance, children’s verbal and non-verbal abilities prior to school start have been shown to have a mediating effect on the relationship between SES and school performance^20^ whilst working memory has been found to partially mediate correlations between parental education and children’s math abilities^21^. Executive functions and its relationship with both SES and academic indices have been investigated at various ages, often specifically with respect to children’s mathematical abilities^21–26^. Activities in young children’s home environment aimed at providing cognitive stimulation, such as caregiver’s involvement, have been shown to promote children’s language abilities which in turn predicted later academic achievement^27^. However, several other studies do not confirm significant effects of SES on individual differences in school achievement once intelligence is factored in refs. ^28–30^. The relationship between SES and success in school is complex and most realistically evaluated when various influences like subject areas, school types and other non-cognitive variables, such as ethnicity, gender, personality, or motivational factors, are also considered^2,31–35^. Overall, intelligence seems to be the strongest and most reliable predictor of academic performance^36^, although additional factors, both within and beyond the individual, also come into play.

While there is compelling evidence that low SES can compromise a child’s educational success, there seem to be many cases in which this potentially adverse dynamic does not take effect, where disadvantaged children do well in school despite perhaps having the odds stacked against them. This phenomenon, commonly referred to as academic resilience in the literature^35,37^, postulates that some individuals succeed in their academic career despite coming from unfavorable conditions, including but not restricted to low SES. A conceptually related term is academic buoyancy^38^ which is concerned with academic resilience in a more day-to-day sense of the word. It is defined as a student’s capacity to cope with everyday challenges and hardships at school in the absence of socioeconomic or other types of disadvantaged living conditions. Various potentially protective factors have been discussed in this regard, though it remains uncertain what exactly it is that makes some people less prone to suffer negative consequences from the adverse conditions they were born into^35^ than others. Psychological traits that have been found to predict academic resilience are self-efficacy, control, planning, low anxiety, as well as persistence^39^. Other traits and attitudes that have been associated with academic resilience are students’ confidence in reading and math as well as a sense of belonging to their school^40,41^. In addition, factors relating to children’s home, classroom, and school environment, can affect academic resilience. For instance, early supportive literacy activities prior to primary school start, positive teacher attitude towards their students’ performance, low levels of bullying, safe and orderly environments and a teaching style that is focused on comprehension and reflection^40,41^. Academic resilience in turn appears to be linked to school enjoyment, class participation and overall self-esteem^39^. Cross-national research found differences in the mechanisms underlying academic resilience between native and immigrant students with a foreign background being associated with overall lower academic resilience^42,43^. Typically, adverse life events with respect to academic resilience pertain to the individual level. The Covid-19 pandemic on the other hand, has recently induced life-event stress on a global scale and has been investigated with respect to different risk and resilience factors affecting students’ success in coping with the hardships of isolation during lockdown^44,45^.

The aim of the present work is to add to this emergent area of research by testing whether the frequently demonstrated interplay between socioeconomic background and school performance affects children in different ways. Evaluating if interindividual differences in cognitive performance and socioeconomic variables interact with one another in their effect on grades could yield important additional clues about what else fosters academic resilience beyond the personality, behavioral, and environmental factors discussed above. Intelligence and cognitive ability have robustly been associated with academic achievement across various subjects^46–49^ as well as a wide range of other performance indicators in both educational and professional settings^50,51^. At the same time, children’s socioeconomic status has been tied to several cognitive abilities, most prominently executive function, and language^52–59^ as well as academic performance^1–4^. While effects of cognitive abilities on academic achievement, and, in turn, of SES on both the former and the latter are well documented, interactions between the three are less explored. Earlier work has investigated whether cognitive ability affects the relationship between SES and school performance on a country level and has not confirmed such effects^60^. By contrast, in this work, we seek to determine whether the relationship between socioeconomic background and school achievement will differ as a function of cognitive ability on an individual level. The present analysis offers additional value by drawing on academic achievement data from two timepoints. First, we assess correlations between SES and self-reported grades in a subsample of 9-to-11-year-old participants in the Adolescent Brain Cognitive Development (ABCD) study. Next, we reinvestigate those associations between socioeconomic background variables and school performance, this time factoring in domain-general cognitive performance to determine whether one’s cognitive ability has a bearing on how socioeconomic background relates to school achievement. To get an idea of how the relationship between SES and grades unfolds over time, school performance data from two timepoints (i.e., 2-year and 3-year-follow-up following baseline assessment) are used for modeling.

## Results

### Associations between SES and grades at 2-year and 3-year-follow-up

We assessed associations between SES and school performance by fitting separate ordinal logistic regression models for each of the two timepoints, using grades as dependent variable and parental education, income-to-needs ratio, and ADI as predictors. The patterns between the different socioeconomic variables and grades were very similar at both the 2-year and the 3-year follow-up. For both models, the odds ratio for obtaining a higher grade at school increased with increasing levels of each of the three SES measures. More affluent neighborhoods receive lower ADI scores, hence an odds ratio smaller than 1 for ADI indicates a positive relationship between living in a wealthier neighborhood and obtaining a higher grade. See Tables 1 and 2 for an overview of regression outcomes for all predictors along with odds ratios (and 95% confidence intervals) at 2- and 3-year follow-up.

**Table 1:** Odds ratios (with 95% confidence intervals) for reporting higher grades dependent on socioeconomic background at 2-year follow-up (*N* = 5001)

Predictor	*β*	SE	*z*	*p*	OR (with 95% CI)
Parental education	0.26	0.03	7.48	0.00	1.29 (1.21, 1.38)***
ITN	0.31	0.04	7.58	0.00	1.37 (1.26, 1.49)***
ADI (percentiles)	−0.10	0.03	−2.94	0.00	0.91 (0.85, 0.97)**

*SE* standard error, *OR* odds ratio.

**p < 0.01, ***p < 0.001.

**Table 2:** Odds ratios (with 95% confidence intervals) for reporting higher grades dependent on socioeconomic background at 3-year follow-up (*N* = 5001)

Predictor	*β*	SE	*z*	*p*	OR (with 95% CI)
Parental education	0.30	0.03	8.91	0.00	1.34 (1.26, 1.43)***
ITN	0.31	0.04	7.66	0.00	1.36 (1.26, 1.47)***
ADI (percentiles)	−0.11	0.03	−3.45	0.00	0.89 (0.84, 0.95)***

*SE* standard error, *OR* odds ratio.

***p < 0.001.

### Influence of SEs on grades over time

Following the individual models for each timepoint, we conducted post hoc analyses modeling interactions between the three SES indices and timepoint, which corroborated significant main effects of parental education, income-to-needs ratio, and ADI on predicted grade. In addition, a significant effect of timepoint emerged, implying that the odds for obtaining a good grade were higher at the 2-year compared to the 3-year follow-up. Interactions between the three SES metrics and time, on the other hand, were not statistically significant effects. See Table 3 for details regarding the regression estimates.

**Table 3:** Odds ratios (with 95% confidence intervals) for reporting higher grades as a function of SES and time (*N* = 5001)

Predictor	*β*	SE	*z*	*p*	OR (95% CI)
Parental education	0.26	0.03	7.50	0.00	1.29 (1.21, 1.38)***
ITN	0.32	0.04	7.60	0.00	1.37 (1.26, 1.49)***
ADI (percentiles)	−0.10	0.03	−2.95	0.00	0.91 (0.85, 0.97)**
Timepoint	−0.20	0.04	−4.55	0.00	0.82 (0.76, 0.89)***
Timepoint*Education	0.04	0.05	0.81	0.42	1.04 (0.95, 1.14)
Timepoint*ITN	−0.01	0.06	−0.17	0.86	0.99 (0.88, 1.10)
Timepoint*ADI	−0.01	0.05	−0.31	0.76	0.99 (0.89, 1.08)

**p < 0.01, ***p < 0.001.

### Associations between SES and grades at 2-year and 3-year-follow-up as a function of cognitive ability

We proceeded to fit two additional ordinal regression models, one for each timepoint, to examine whether the overall effect of SES on grades changes as a function of children’s cognitive ability. The results, shown in Tables 4 and 5, indicate that at both timepoints cognitive ability and parental education interact with one another in their effect on the odds of reporting higher grades in school. Neighborhood deprivation (ADI) on the other hand was no longer a significant predictor of grades when cognitive ability was factored into the models. Income-to-needs ratio (ITN) was a significant predictor of grade both at the 2-year and the 3-year follow-up, implying that increases in income were associated with higher odds of reporting a better grade at both timepoints. Pairwise comparisons of the models for each of two timepoints indicated superior model fit when cognitive abilities were included in the models (i.e., better fit of the models reported in Tables 4 and 5 compared to the models reported in Tables 1 and 2; *p* < 0.001 in both cases).

**Table 4:** Odds ratios (with 95% confidence intervals) for reporting higher grades at school at 2-year follow-up dependent on interactions between socioeconomic background and cognitive performance (*N* = 5001)

Predictor	*β*	SE	*z*	*p*	OR (95% CI)
Parental education	0.20	0.04	5.22	0.00	1.22 (1.13, 1.32)***
ITN	0.24	0.04	5.65	0.00	1.27 (1.17, 1.39)***
ADI (percentiles)	−0.06	0.04	−1.55	0.12	0.94 (0.88, 1.02)
Composite cognition	0.54	0.04	15.08	0.00	1.72 (1.60, 1.84)***
Cognition*Education	0.08	0.04	2.10	0.04	1.08 (1.00, 1.16)*
Cognition*ITN	0.03	0.04	0.65	0.52	1.03 (0.94, 1.12)
Cognition*ADI	−0.06	0.04	−1.78	0.07	0.94 (0.88, 1.01).

*SE* standard error, *OR* odds ratio.

. indicates *p* < 0.1, *p < 0.05, ***p < 0.001.

**Table 5:** Odds ratios (with 95% confidence intervals) for reporting higher grades at school at 3-year follow-up dependent on interactions between socioeconomic background and cognitive performance (*N* = 5001)

Predictor	*β*	SE	*z*	*p*	OR (95% CI)
Parental education	0.25	0.04	6.79	0.00	1.29 (1.20, 1.69)***
ITN	0.24	0.04	5.93	0.00	1.27 (1.18, 1.38)***
ADI (percentiles)	−0.07	0.04	−1.94	0.05	0.93 (0.87, 1.00).
Composite cognition	0.46	0.03	13.39	0.00	1.58 (1.48, 1.69)***
Cognition*Education	0.08	0.03	2.31	0.02	1.08 (1.01, 1.16)*
Cognition*ITN	0.03	0.04	0.66	0.51	1.03 (0.94, 1.12)
Cognition*ADI	−0.03	0.03	−0.90	0.37	0.97 (0.91, 1.04)

*SE* standard error, OR odds ratio.

. indicates *p* < 0.1, *p < 0.05, ***p < 0.001.

## Discussion

The idea behind the present work was to determine whether cognitive ability can contribute to boost academic resilience through its joint effect with socioeconomic status on school performance. More precisely, we wanted to test whether higher cognitive ability may have the potential to protect children from known adverse effects of low socioeconomic status on educational attainment. A better understanding of whether cognitive skills contribute to offsetting known relationships between SES and school performance, would be a valuable starting point for targeted interventions designed to lower the risk of long-term negative consequences for children growing up under socioeconomically disadvantaged conditions.

Firstly, our analysis revealed that at two timepoints, parental education, income-to-needs ratio and neighborhood disadvantage were significant predictors of grades before factoring in cognitive ability—in line with existent literature. At both the 2-year and 3-year follow-up, the odds for reporting higher grades increased with the level of parental education, income-to-needs ratio, and lower levels of neighborhood deprivation, respectively, with larger effect sizes for the first two compared to the latter. This mirrors previous work on the ABCD sample that found the effect of parental education and income on children’s intelligence to be twice as big as neighborhood quality^61^. Similarly, earlier findings from the same cohort^62^ reported that among the different SES indices, household income was most strongly associated with composite cognition scores. Parent education and neighborhood deprivation also correlated with cognition, although the effects were marginal once income was controlled for. In the study, the link between household income and cognition was especially strong for the crystallized component of cognition, suggesting that low income might be particularly detrimental for the development of language-related abilities^62^ as has been demonstrated before, already long before school entry^63^.

Secondly, the present analysis revealed an interaction between parental education and cognition at both timepoints, suggesting that children with better cognitive abilities benefit more from having well-educated parents than children with weaker cognitive performance. While the interaction was statistically significant, the size of the effect was small, making it hard to conceive of it as having practical relevance, even on a populational level. We conjecture that it is more likely that both cognitive ability and parental education exert substantial individual influence on children’s grades and that a potential interplay between the two is more complex than what can be captured by a simple regression model. This finding echoes earlier work at the country level in which the relationship between school performance and SES was not affected by cognitive ability^60^. The effect sizes we observed were modest and did not differ considerably between the 2-year and the 3-year follow-up assessment.

Thirdly, when directly examining the impact of SES on grades over time, interaction effects between time and each of the three SES indices fell short of reaching statistical significance. Considering that the requirements students face in school gradually increase, one could reason that coming from a privileged background with possibly more generous resources for intellectual stimulation, guidance, and support, becomes more beneficial the more difficult the demands in school become. It goes without saying that socioeconomic disadvantage does not automatically go hand in hand with a lack of cognitive stimulation in a child’s home environment on an individual level. Previous data indicating only a weak relationship between SES and academic achievement in a higher education setting^64,65^ contradicts the assumption that the impact of SES on academic performance increases over time. One conceivable explanation is that, with age, children gradually acquire metacognitive strategies that help them to remain on track and pursue and attain academic goals beyond what their socioeconomic background alone would have set them up for. A meta-analytic review evaluating the long-term effects of interventions to teach students learning strategies supports this idea by indicating greater long-term benefits of strategy instructions for students with low SES^66^. Once data from additional timepoints becomes available, it will be interesting to examine whether the lack of relationship between SES and time is confirmed further down the developmental line as the ABCD participants transition from childhood further into adolescence.

Comparing socioeconomic economic status between the children that reported top grades and those that did not reveals that overall, the patterns resemble one another in both groups. However, the higher performing group is socioeconomically better off, with a larger share of children coming from higher income households with more highly educated parents compared to the children that report weaker grades. The children in that group are also more likely to live in neighborhoods that offer favorable conditions in terms of factors such as housing, employment, and education. Even though the design of the present work does not allow for a differentiation of cause and effect, our findings clearly highlight systematic differences between children whose manifestations tend to co-occur. In this case that means that children who report better grades in school on average perform better on a neurocognitive test battery and are more likely to be socioeconomically better off. This finding points towards the existence of the notorious “Matthew effect”^67^ in an educational context. The term was first coined in the 1960s, originally to describe the phenomenon that reputable scientists are more likely to receive additional recognition for their work than an unknown colleague despite comparable achievements. The term has since been applied in a variety of contexts in which initial resources predetermine future gains, often trenchantly summarized as “the rich get richer, and the poor get poorer”. While our observational study design does not permit causal statements, the present work corroborates clearly that growing up in favorable socioeconomic circumstances likely occurs along with additional positive outcomes.

The flipside of having a large dataset available is the observational nature of the ABCD Study® which rules out causal and more directional statements about the interdependences between cognitive ability, different indicators of SES and school performance. Despite this undeniable drawback, we argue that the present study is important in that it points towards associations between socioeconomic status and grades in a large, longitudinal sample of children that are persistent over time. This is evidently fraught with problems considering that the central idea behind using standardized tests and grading in educational settings is to obtain an objective and unbiased assessment of children’s true level of ability—and not factors beyond their control, such as their societal or financial status. It is not surprising that variations in cognitive capacity are not sufficient to explain differences in the relationship between socioeconomic background and performance in school. Additional variables, for instance personality and attitude factors also exercise influence in a way that likely varies both within and between individuals. The already complex situation is aggravated by the fact that socioeconomic variables are typically highly correlated with each other. A person that is well-educated tends to earn more than someone with fewer years of formal education and has a higher likelihood of living in affluent neighborhoods, making it difficult to disentangle unique contributions of each of these to educational performance. Despite multicollinearity among predictor variables though, we still show unique and robust contributions of the three SES indicators that were investigated.

Participants in the ABCD Study® were recruited via their respective schools across the United States, resulting in a nested data structure with multiple children attending the same school. It is possible that there are systematic differences between the grades reported by children going to different schools. For example, children attending schools with more resources, more highly qualified teachers or smaller class sizes may have better chances of obtaining good grades than their peers from schools that are comparably less well-off. It is also conceivable that the level of difficulty of tests and grading practices might differ between schools. Unfortunately, information regarding which school participants attend is not available for a large proportion of the data. Therefore, we were not able to statistically account for the nested structure of the data in our analyses. Looking ahead, a valuable supplement to the present study would be work assessing the robustness of our results when school-specific variations are taken into account. There is also some uncertainty as to how well the children’s self-reported performance in school maps onto their true level of academic achievement and a certain extent of discrepancy cannot be ruled out. Having our analysis rest on children’s self-reported grades is far from optimal, since the evidence regarding their reliability is mixed^68,69^. However, the same could be argued about parental estimates about their own children, which have been shown to be biased in different contexts^70,71^. Findings derived from self-reports, regardless of whether they come from the children themselves or their parents, can never be interpreted with the same confidence as would be the case for objective measures, but in the absence of school transcripts, we have no choice but to make do with what is available as well as possible.

While the ABCD sampling strategy was designed to yield a participant pool that is representative of the general population in the United States, that does not automatically imply that the final sample meets this goal across all variables^72^. The fact that the ABCD sample is composed of volunteers likely introduces systematic bias since individuals that opt to participate in a research study can be presumed to differ in more than one way from those that do not^72^. Since the ABCD sites had to be located where neuroimaging equipment and expertise were available, the sample is biased towards metropolitan areas, underrepresenting individuals from rural regions^72^. The ABCD cohort is socioeconomically skewed with disproportionately many parents being highly educated and economically well-off. In the present sample, 46% of children live in a household with an income of at least 100,000 USD per year and, by more than a factor of two, 100,000 through 199,000 USD is the most frequent household income bracket, reported for every third child in this sample. By comparison, according to the U.S. Census Bureau, the median household income during 2017 amounted to 60,336 USD (https://www.census.gov/content/dam/Census/library/publications/2018/acs/acsbr17-01.pdf, last accessed on 07 September 2023). Similarly, nearly 61% of parents in the current sample have completed at least a bachelor’s degree compared to just under 40% of US adults aged 25 and older overall according to the U.S. Census Bureau (https://www.census.gov/newsroom/press-releases/2022/educational-attainment.html, last accessed on 19 December 2022)—giving the impression that low SES families are inadequately represented in this study. Consequently, the present study’s explanatory power when it comes to links between low socioeconomic status and grades is limited and further research is needed to verify if the trends, we observed among the higher SES levels here apply to socioeconomically disadvantaged children in a comparable way.

In line with earlier work, the present study demonstrates significant effects of three different indices of socioeconomic status on children’s self-reported grades in a large sample across two timepoints. In addition, our results point towards parental education and children’s cognitive performance interacting in their effect on children’s self-reported performance in school. A child’s odds to do exceptionally well academically increased the more educated and financially well-off their parents were, with steeper slopes for children with better cognitive performance. Since the size of this effect was negligible, our study does overall not provide strong evidence for robust associations between socioeconomic status and grades as a function of cognitive ability. The effect sizes we found were generally modest which is not necessarily surprising. It makes sense that the combined effect of various factors, located both within and outside the individual, determines a child’s success in school and that among these, socioeconomic background and cognitive abilities can only explain variance in grades up to a certain point. Still, this work illustrates that across time and levels of cognitive capacity, children’s grades are in parts influenced by their socioeconomic background. This is in line with previous literature and calls on teachers, policy makers, and educational researchers to continue to strive towards creating school environments in which a child’s chances at distinguishing themselves academically are controlled entirely by them, rather than being determined by how wealthy and well-educated their parents are. Anyone who is in some way involved with children’s schooling is called upon to be mindful of the possibility of systematic socioeconomic disadvantage and to ensure that optimal learning conditions are created suitable for each child’s individual situation. Especially in cases where the availability of intellectual stimulation and encouragement in the home environment may be limited, resources need to be readily available outside children’s homes and children should actively be encouraged to take advantage of them.

## Methods

### Participants

A subset of participants from the publicly available data from the *Adolescent Brain Cognitive Development* (ABCD) study was formed based on the following inclusion criteria: complete data for the neurocognitive assessment at baseline, information on the children’s estimation of their overall performance in school in the last year for both the 2- and 3-year-follow-up evaluation, and baseline data on the parent’s educational level, household size, and combined household income during the past year as well as data on neighborhood SES. This yielded a sample of 5001 children (mean age = 9.98 years, SD = 0.6), 2656 of which were boys (53%) and 2345 girls. All data that was used for this analysis is available as part of the ABCD Annual Release 4.0 (10.15154/1523041; last accessed on December 26, 2023).

### Materials

#### Cognitive performance

To assess different components of cognitive ability, the ABCD Study® makes use of seven measures, all of which are included in the NIH Toolbox Cognition Battery (https://www.healthmeasures.net/explore-measurement-systems/nih-toolbox/intro-to-nih-toolbox/cognition; accessed on December 26, 2023). These tests tap into attention, executive functioning, episodic memory, working memory, language, and processing speed. They are iPad-based and administered in English. Details about the individual tasks have been described elsewhere^19,73^. All cognitive data used in the present analyses stems from the ABCD baseline assessment. As an indicator of global cognitive ability, we use a standardized composite cognition score that summarizes a child’s performance across all seven cognitive tasks and is available as part of the tabulated data releases provided by the ABCD. Both age-corrected and non-age-corrected standard scores are available within the ABCD dataset. Due to the narrow age range in the sample, we opted to base our analyses on the uncorrected scores.

#### SES

Socioeconomic status is a comprehensive, multifaceted concept and can be operationalized in many ways, using either individual variables or compound measures derived from these. Educational attainment, occupation, and income are variables that are typically taken into consideration when characterizing an individual’s socioeconomic status. SES measures can be divided into area and household-based metrics, where the latter characterizes the individual’s immediate home environment, whereas the former gives a more general indication about the conditions in the region that their home is situated in in terms of factors such as public finance, safety, employment, or housing. The ABCD participant’s socioeconomic background is described using both these sources. Household-based metrics include parental education, employment status, and income. Areal metrics include the so-called Area Deprivation Index (ADI), indicating the level of socioeconomic disadvantage at the participant’s place of residence. The analyses in the present study are based on three SES indicators: parental education, income-to-needs ratio, and neighborhood deprivation. Parental education specifies the highest level of school/degree that was obtained by the parent. The response scale ranges between 0 to 21 (where 0 corresponds to never having participated in any form of formal education and 21 is equivalent to having obtained a PhD) where numerical values approximately match the number of years that someone spent getting formally educated. Parental education is treated as continuous variable in this analysis. In line with previous work^74,75^, we computed income-to-needs ratio (ITN) to quantify participants’ financial circumstances. To calculate this metric, we divided the median value for the household income bracket that was reported for the child by the U.S. Federal Poverty Guidelines for 2017 for the respective household size. Thus, an income-to-needs ratio of 1 is equivalent to living at poverty level. Values smaller than 1 imply living conditions below poverty level and values above 1 imply a proportional surplus of financial means compared to poverty level. Neighborhood SES is quantified by means of the Area deprivation index (ADI), where lower scores suggest wealthier neighborhoods and higher scores indicate more deprived areas. As per recommendation from the ABCD consortium, we used ADI national percentiles for our analyses. Both ITN and ADI are treated as continuous variables. All SES information that was used for the present work was collected as part of the ABCD Study®’s baseline assessment.

#### Grades

The ABCD Study® does not provide information on the participants’ grades or other performance indicators directly from their schools. Instead, both the children and their parents are asked to provide an overall rating of how well they had done at school during the last year. While visual comparison of the distributions of self-reported grades by the children and their parents indicates a high overlap between both estimates, Wilcoxon signed-rank tests suggest significant differences between the distributions at both timepoints. However, since the effect sizes of these were negligible (*r* = 0.1 at the 2-year-follow-up and *r* = 0.05 at the 3-year-follow-up) we only included the children’s self-reported data in our analyses. Grade estimates as provided by the ABCD Study® range between 1 (corresponding to A+) and 12 (corresponding to a failing grade). For this work, we recoded the grade variable (in accordance with information from the ABCD Study®), resulting in the five levels A, B, C, D, and Fail.

### Procedure

To assess the strength of association between SES variables and grades, we first fit two separate ordinal logistic regression models, one for each timepoint. Grades at the respective timepoint served as dependent variable and parental education, income-to-needs ratio, and neighborhood deprivation were used as predictors. Next, we expanded the initial models by including three interaction terms consisting of composite cognition and parental education, income-to-needs ratio, and neighborhood deprivation, respectively. Lastly, we performed a post hoc regression analysis to test the effect of the three SES indices on grade over time by including interaction terms between parental education, income-to-needs ratio, respectively, neighborhood deprivation and timepoint. All statistical tests were performed in R (version 4.2.2)^76^ and all predictor variables were scaled prior to analysis. The parallel regression assumption, as assessed by the Brant test, was met for all models.

### Reporting summary

Further information on research design is available in the Nature Research Reporting Summary linked to this article.

### Supplementary information

Reporting summary

## Data Availability

Data used in the preparation of this article (10.15154/1519007; accessed on 24 February 2024) were obtained from the Adolescent Brain Cognitive Development^SM^ (ABCD) Study (https://abcdstudy.org, accessed on 24 February 2024), held in the NIMH Data Archive (NDA).
