# Supplementary material for: Interplay of socioeconomic status, cognition, and school performance in the ABCD sample
Source: NPJ Sci Learn. 2024 Mar 11;9:17. doi: 10.1038/s41539-024-00233-x (PMC10928106; doi:10.1038/s41539-024-00233-x)
Supplement: Supplementary file 1 — Reporting summary [file 41539_2024_233_MOESM1_ESM.pdf]

nature portfolio

Corresponding author(s): Lara Langensee

Last updated by author(s): Feb 24, 2024

# Reporting Summary

Nature Portfolio wishes to improve the reproducibility of the work that we publish. This form provides structure for consistency and transparency in reporting. For further information on Nature Portfolio policies, see our Editorial Policies and the Editorial Policy Checklist.

## Statistics

For all statistical analyses, confirm that the following items are present in the figure legend, table legend, main text, or Methods section.

| n/a | Confirmed | |
|---|---|---|
| ☐ | ☒ | The exact sample size (*n*) for each experimental group/condition, given as a discrete number and unit of measurement |
| ☐ | ☒ | A statement on whether measurements were taken from distinct samples or whether the same sample was measured repeatedly |
| ☐ | ☒ | The statistical test(s) used AND whether they are one- or two-sided<br>*Only common tests should be described solely by name; describe more complex techniques in the Methods section.* |
| ☐ | ☒ | A description of all covariates tested |
| ☒ | ☐ | A description of any assumptions or corrections, such as tests of normality and adjustment for multiple comparisons |
| ☐ | ☒ | A full description of the statistical parameters including central tendency (e.g. means) or other basic estimates (e.g. regression coefficient) AND variation (e.g. standard deviation) or associated estimates of uncertainty (e.g. confidence intervals) |
| ☐ | ☒ | For null hypothesis testing, the test statistic (e.g. *F*, *t*, *r*) with confidence intervals, effect sizes, degrees of freedom and *P* value noted<br>*Give P values as exact values whenever suitable.* |
| ☒ | ☐ | For Bayesian analysis, information on the choice of priors and Markov chain Monte Carlo settings |
| ☒ | ☐ | For hierarchical and complex designs, identification of the appropriate level for tests and full reporting of outcomes |
| ☐ | ☒ | Estimates of effect sizes (e.g. Cohen's *d*, Pearson's *r*), indicating how they were calculated |

*Our web collection on statistics for biologists contains articles on many of the points above.*

## Software and code

Policy information about availability of computer code

| | |
|---|---|
| Data collection | Data used in this manuscript is from the ABCD study, Annual Release 4.0 (http://dx.doi.org/10.15154/1523041) |
| Data analysis | R version 4.2.2, standard analysis using the clm() function from the ordinal package |

For manuscripts utilizing custom algorithms or software that are central to the research but not yet described in published literature, software must be made available to editors and reviewers. We strongly encourage code deposition in a community repository (e.g. GitHub). See the Nature Portfolio guidelines for submitting code & software for further information.

## Data

Policy information about availability of data

All manuscripts must include a data availability statement. This statement should provide the following information, where applicable:

- Accession codes, unique identifiers, or web links for publicly available datasets
- A description of any restrictions on data availability
- For clinical datasets or third party data, please ensure that the statement adheres to our policy

Data used in the preparation of this article (http://dx.doi.org/10.15154/1519007; accessed on 24 February 2024 were obtained from the Adolescent Brain Cognitive Development (ABCD) Study (https://abcdstudy.org, accessed on 24 February 2024), held in the NIMH Data Archive (NDA).

## Research involving human participants, their data, or biological material

Policy information about studies with human participants or human data. See also policy information about sex, gender (identity/presentation), and sexual orientation and race, ethnicity and racism.

| | |
|---|---|
| Reporting on sex and gender | Sex and gender were not included in the present analysis, because they were not of interest for the research question. Data on sex is reported only in the form of descriptive statistics on the sample. Data about assigned sex at birth was collected via parent questionnaire. |
| Reporting on race, ethnicity, or other socially relevant groupings | Parental education and income information as reported by parents and information on neighborhood deprivation (from census data) are used as dependent variables in the analyses. |
| Population characteristics | See above. |
| Recruitment | Participants for the ABCD studies were recruited via schools, see Garavan et al. 2018 for details. |
| Ethics oversight | Ethical review and approval for all data collected within the scope of the ABCD study comes either from a central Institutional Review Board (IRB) located at the University of California, San Diego, or, in the case of some sites, from local IRBs. Written informed consent was obtained from parents/guardians and assent from the participating children. |

Note that full information on the approval of the study protocol must also be provided in the manuscript.

# Field-specific reporting

Please select the one below that is the best fit for your research. If you are not sure, read the appropriate sections before making your selection.

☐ Life sciences ☒ Behavioural & social sciences ☐ Ecological, evolutionary & environmental sciences

For a reference copy of the document with all sections, see nature.com/documents/nr-reporting-summary-flat.pdf

# Behavioural & social sciences study design

All studies must disclose on these points even when the disclosure is negative.

| | |
|---|---|
| Study description | Ordinal logistic regression analysis using data from the observational, longitudinal ABCD Study |
| Research sample | A subset of 5001 9-to-11-year-old children from the ABCD sample which was collected aiming to be representative of the characteristics of the general population in terms of important demographics. |
| Sampling strategy | The ABCD Study employed a stratified sampling strategy to ensure representativeness of the sample in terms of gender, race, ethnicity, SES, and urbanicity. Further details are available in Garavan et al. 2018. We used the complete ABCD dataset and selected all participants that did not meet any of our exclusion criteria (see below), this left us with a sample of N=5001. |
| Data collection | Grades were reported by the children and their parents, information about SES was reported by the parents, neighborhood deprivation is based on census data, the cognitive tests were administered on an iPad with an experimenter present. |
| Timing | ABCD data collection commenced in 2017 and is planned to continue over the course of 10 years with different types of data collected at regular intervals (of different length, depending on the type of data). More details regarding design considerations of the ABCD Study are available in Garavan et al. 2018. |
| Data exclusions | We excluded participants that had missing cognitive or SES data or that did not have grade information available for both 2- and 3-year follow-up. This criteria were established prior to the analysis. |
| Non-participation | All details about design considerations for the ABCD study are found in Garavan et al. 2018. |
| Randomization | Participants were not allocated to groups for the analyses. |

# Reporting for specific materials, systems and methods

We require information from authors about some types of materials, experimental systems and methods used in many studies. Here, indicate whether each material, system or method listed is relevant to your study. If you are not sure if a list item applies to your research, read the appropriate section before selecting a response.

## Materials & experimental systems

| n/a | Involved in the study |
|---|---|
| ☒ | ☐ Antibodies |
| ☒ | ☐ Eukaryotic cell lines |
| ☒ | ☐ Palaeontology and archaeology |
| ☒ | ☐ Animals and other organisms |
| ☒ | ☐ Clinical data |
| ☒ | ☐ Dual use research of concern |
| ☒ | ☐ Plants |

## Methods

| n/a | Involved in the study |
|---|---|
| ☒ | ☐ ChIP-seq |
| ☒ | ☐ Flow cytometry |
| ☒ | ☐ MRI-based neuroimaging |

# Plants

| | |
|---|---|
| Seed stocks | N/A |
| Novel plant genotypes | N/A |
| Authentication | N/A |
